# Public knowledge and acceptance of genetic testing for disability-related diseases in pharmacies: A cross-sectional study from Saudi Arabia

**DOI:** 10.1097/MD.0000000000043866

**Published:** 2025-08-15

**Authors:** Nasser M. Alorfi, Saad M. Wali, Ahmed M. Ashour, Fahad S. Alshehri, Ohood K. Almuzaini, Maan H. Harbi, Mohammed M. Aldurdunji, Mustfa Faisal Alkhanani, Reem Hasaballah Alhasani, Shaker T. Alsharif, Mohammed S. Alharthi, Nasser M. Aldekhail

**Affiliations:** a Pharmacology and Toxicology Department, College of Pharmacy, Umm Al-Qura University, Makkah, Saudi Arabia; b King Salman Center for Disability Research, Riyadh , Saudi Arabia; c Pharmaceutical Practices Department, College of Pharmacy, Umm Al-Qura University, Makkah, Saudi Arabia; d Biology Department, College of Sciences, University of Hafr Al Batin, Hafr Al Batin, Saudi Arabia; e Department of Biology, Faculty of Science, Umm Al-Qura University, Makkah, Saudi Arabia; f Pharmaceutical Sciences Department, College of Pharmacy, Umm Al-Qura University, Makkah, Saudi Arabia; g Department of Clinical Pharmacy, College of Pharmacy, Taif University, Taif, Saudi Arabia; h Department of Pharmacy, College of Pharmacy, Nursing and Medical Sciences, Riyadh Elm University, Riyadh, Saudi Arabia.

**Keywords:** acceptance, disability, genetic testing, pharmacy, public perception

## Abstract

Genetic testing offers significant potential for early detection, diagnosis, personalized treatment, and preventive strategies for disability-related diseases. Pharmacies, as accessible healthcare hubs, can play a pivotal role in delivering genetic testing services. However, public acceptance and awareness remain limited, particularly in Saudi Arabia. This study aims to evaluate the public knowledge, perceptions, and acceptance of genetic testing for disability-related diseases in Saudi Arabian pharmacies. A cross-sectional survey was conducted with 475 participants recruited via convenience sampling on social media. Data were collected using a structured questionnaire assessing demographics, knowledge, perception, and acceptance of pharmacy-based genetic testing. Statistical analyses, including descriptive statistics, *t*-tests, ANOVA, and regression modeling, were performed. While 57.6% of participants reported being somewhat aware of genetic testing, only 9.1% considered themselves very knowledgeable. Perceived benefits included early detection (84.4%) and preventive measures (64.4%). Approximately 42.5% felt comfortable with pharmacy-based genetic testing, but concerns regarding pharmacist expertise (57.7%) and test accuracy (50.7%) were prominent barriers. Acceptance was influenced significantly by age, gender, education level, and healthcare background. Despite moderate awareness and acceptance of genetic testing services in pharmacies, significant barriers remain. Addressing public concerns, improving pharmacist training, and enhancing collaborative care models are essential for successful integration of genetic testing into pharmacy practice.

## 1. Introduction

Genetic testing has emerged as a transformative tool in modern medicine, providing critical insights into an individual’s predisposition to various genetic conditions.^[1,2]^ For diseases classified as disabilities, such as multiple sclerosis, Parkinson disease, rheumatoid arthritis and cerebral palsy, genetic investigations could offer the potential for early detection, diagnosis, personalized treatment plans, and informed decision-making regarding management and prevention strategies.^[3–8]^ They would also be valuable for other disabilities like visual and hearing impairment, mental health disorders, learning and physical disability, speech or language impairment, and other disabilities that require special care. Each of these disabilities is different for everyone in terms of its severity and the possibility of treatment.

With the growing burden of these diseases in Saudi Arabia, genetic testing represents a significant advancement in healthcare. According to the 2022 data published by the General Authority for Statistics in Saudi Arabia, approximately 1.35 million individuals were identified as having at least 1 mild physical difficulty or disability, which constitutes 4.2% of the total population. Among these, 1.112 million were Saudi nationals, accounting for 5.9% of the Saudi population, while 238,000 were non-Saudis, representing 1.8% of the non-Saudi population.^[9]^ When analyzed by gender, 765,000 males (3.9% of the total male population) and 585,000 females (4.7% of the total female population) were affected. Of this group, 588,000 individuals had at least 1 disability, and 891,000 reported experiencing one or more mild physical difficulties, equivalent to 2.8% of the overall population. However, the successful implementation of genetic testing services depends largely on public awareness and acceptance, which remain underexplored, particularly in the context of community pharmacies.

Pharmacies are increasingly being recognized as key access points for healthcare, particularly in rural and underserved communities where access to primary healthcare facilities may be limited. In Saudi Arabia, pharmacies serve as easily accessible healthcare hubs, offering not only prescription medications but also consultations and health services.^[10,11]^ Expanding the role of pharmacies to include genetic testing services for disability-related diseases could make these services more accessible to the

public. However, understanding how the public perceives such a shift is crucial for its success. Public knowledge of genetic testing, awareness of its benefits and limitations, and concerns regarding privacy, costs, and the potential psychological impact of the results play a central role in determining acceptance.

This study aims to reduce the knowledge gap by assessing public awareness and acceptance of genetic testing for disability-related diseases, specifically in the context of pharmacies in Saudi Arabia. By exploring public perceptions, the study will help identify the factors that influence acceptance, including the role of demographic factors such as age, education, and geographic location. Understanding these factors is essential for developing strategies to enhance public engagement and support the integration of genetic testing services into community pharmacies. The findings of this research will not only contribute to the existing body of knowledge but also offer valuable insights for policymakers, healthcare providers, and pharmacists aiming to expand genetic testing services in Saudi Arabia and beyond.

## 2. Methods

A cross-sectional survey was conducted with 475 participants recruited through convenience sampling using the authors’ accounts on the social media platform X, WhatsApp groups, and LinkedIn. Data collection utilized a structured questionnaire with 4 sections: demographics, knowledge of genetic testing, perception of genetic testing in pharmacies, and acceptance levels. The survey included Likert-scale items and open-ended questions.

The reliability of the survey domains was assessed using Cronbach alpha. Construct validity was evaluated through correlations between domains. Descriptive and inferential statistics, including *t*-tests, ANOVA, and regression analyses, were applied to identify predictors of knowledge, perception, and acceptance scores. Ethical approval was obtained from the Biomedical Ethics Committee at Umm Al-Qura University (Approval No. HAPO-02-K-012-2024-11-2329) prior to the study, and informed consent was secured from all the participants.

### 2.1. Sample size

The sample size was calculated via Raosoft Sample Size Calculator.

### 2.2. Survey

The survey was distributed electronically via Google forms through social media as described above. The survey was designed by the authors and validated by 2 academic staff.

### 2.3. Statistical methods

Data analysis was conducted using IBM SPSS version 27 (IBM Corp., Armonk). Descriptive statistics were used to summarize demographic data, providing frequencies and percentages for categorical variables and means with standard deviations for continuous variables. The study assessed 3 primary domains: Knowledge, Perception, and Acceptance of genetic testing.

1. **Reliability analysis**: Cronbach Alpha was calculated for each domain to evaluate internal consistency. Although the Knowledge (α = 0.581) and Acceptance (α = 0.636) domains showed moderate reliability, the Perception domain (α = 0.467) indicated lower consistency. Construct validity was further assessed using Pearson correlation, revealing significant correlations between Perception and Acceptance scores (*R* = 0.214, *P *< .001), supporting domain validity.
2. **Comparison across demographics**: Independent *t*-tests and 1-way ANOVA were employed to examine differences in domain scores across demographic variables, including age, gender, education level, and healthcare background. post hoc analyses (LSD and Games-Howell tests) were used to determine specific group differences, with adjustments for variance heterogeneity where necessary.
3. **Predictive modeling**: Linear regression analysis was conducted to predict Knowledge, Perception, and Acceptance scores based on demographic factors. B values, along with associated *P*-values, were used to determine the strength and significance of predictors such as age, educational level, gender, and healthcare background. Significance thresholds were set at *P* < .05 for all analyses, providing a robust framework for validating associations and influences within the data.

## 3. Results

**Figure 1.**
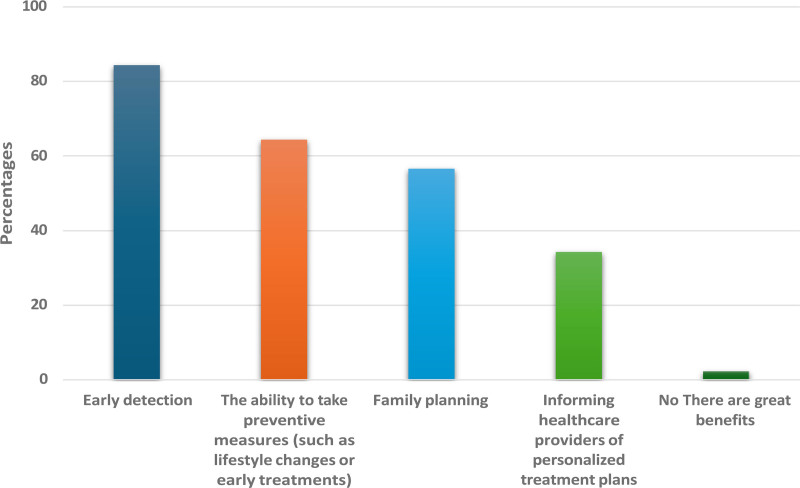
Potential advantages of genetic testing.

A total of 475 participants contributed to the survey. Table 1 outlines their demographic profiles; the majority (52.8%) were aged over 38 years, and 58.1% were male. Educational attainment was predominantly high, with 56.4% holding a university degree, and 66.3% reported a healthcare-related background, which is likely to have influenced their understanding of genetic testing. The potential advantages of genetic testing are shown in Figure 1.

**Table 1:** Characteristics of the 475 study participants.

Demographics		Count	%
Total		475	100.0
Age	18–24	160	33.7
	25–31	27	5.7
	32–38	37	7.8
	More than 38	251	52.8
Gender	Male	276	58.1
	Female	199	41.9
Highest educational level	Less than secondary school	17	3.6
	Secondary school	140	29.5
	University degree	268	56.4
	Postgraduate studies	50	10.5

**Figure 2.**
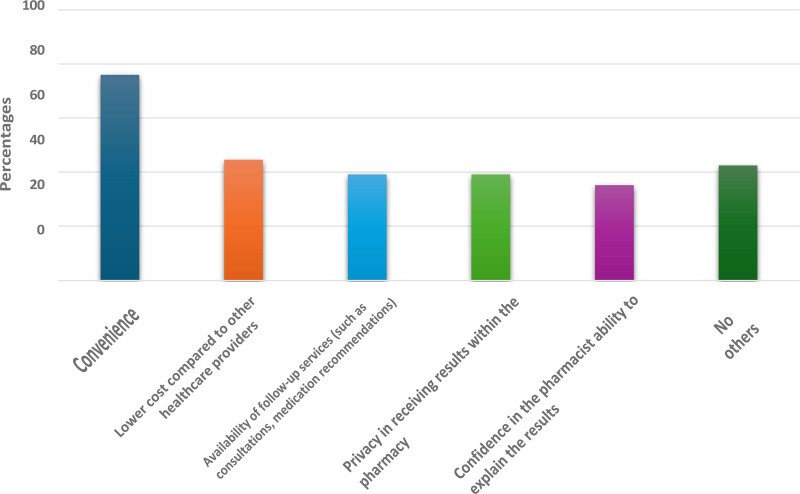
Factors encourage participants to undergo genetic screening for disability-related diseases in a pharmacy.

Knowledge and awareness levels of genetic testing varied among participants, as detailed in Table 2. While 47.6% were “somewhat aware” of genetic screening, only 9.1% considered themselves “very knowledgeable.” Despite limited in-depth knowledge, early detection was recognized by 84.4% as a primary benefit, indicating that awareness of preventive potential exists even among those without extensive familiarity. The preferred sources of genetic counseling are shown in Figure 2.

**Table 2:** Knowledge and awareness of genetic testing.

Variables		Count	%
Total		475	100.0
Do you have a background in healthcare or related fields?	Yes	315	66.3
	No	160	33.7
Have you or any of your family members been diagnosed with a disease that may lead to disability?	Yes, I have been diagnosed	6	1.3
	Yes, a family member has been diagnosed	29	6.1
	No	426	89.7
	Not sure	14	2.9
	Fully aware	45	9.5
How familiar are you with the concept of genetic screening for disease risk assessment?	Somewhat aware	226	47.6
	Not aware	204	42.9
Have you ever undergone genetic testing?	Yes	128	26.9
	No	329	69.3
	Not sure	18	3.8
How would you rate your knowledge of genetic testing?	Very knowledgeable	43	9.1
	Somewhat knowledgeable	233	49.1
	No Knowledge	199	41.9
In your opinion, what are the potential benefits of genetic screening for disability-related diseases?*	Early detection	401	84.4
	Family planning	269	56.6
	Informing healthcare providers of personalized treatment plans	163	34.3
	The ability to take preventive measures (such as lifestyle changes or early treatments)	306	64.4
	There are no great benefits	11	2.3

* Multiple-answer questions result in percentages exceeding 100%.

The perception of genetic screening offered within pharmacies is summarized in Table 3. Here, 42.5% of participants expressed comfort with undergoing genetic screening in a pharmacy, while 39.8% were uncertain. Convenience (76.0%) and affordability (44.6%) were primary motivators for those who were receptive, yet significant concerns were raised regarding pharmacist expertise (57.7%) and test accuracy (50.7%). Notably, female participants were significantly more comfortable with the concept than males (*P *< .001) (see Table 7).

**Table 3:** Perception of Genetic Screening in Pharmacies.

Variables		Count	%
Total		475	100.0
Would you feel comfortable undergoing genetic screening for disability-related diseases in a pharmacy?	Yes	202	42.5
	No	84	17.7
	Not sure	189	39.8
What factors might encourage you to undergo genetic screening for disability-related diseases in a pharmacy?*	Availability of follow-up services (such as consultations, medication recommendations)	186	39.2
	Confidence in the pharmacist’s ability to explain the results	167	35.2
	Convenience	361	76.0
	Lower cost compared to other healthcare providers	212	44.6
	Privacy in receiving results within the pharmacy	186	39.2
	No others	202	42.5
What concerns, if any, do you have about genetic screening for disability-related diseases offered in pharmacies?*	Accuracy of test results	241	50.7
	Cost of the test	153	32.2
	Fear of the results	111	23.4
	Lack of pharmacist’s expertise in genetic testing	274	57.7
	Lack of trust in the technology	79	16.6
	Privacy of genetic data	139	29.3
	No concerns	72	15.2
Would you prefer to receive genetic counseling from a pharmacist if you were screened for a disability-related disease in a pharmacy?	Yes, I prefer that	152	32.0
	No, I prefer to receive consultation from a specialist doctor	242	50.9
	Not sure	81	17.1
If your genetic screening results indicated that you were at an increased risk for a disability-related disease, how likely would it be that you would take preventive measures?	Highly likely	354	74.5
	Maybe	91	19.2
	No at all	30	6.3
Do you think pharmacies should collaborate with specialist physicians to provide comprehensive genetic screening services for disability-related diseases?	Yes	361	76.0
	No	35	7.4
	Not sure	79	16.6
Would you be willing to undergo genetic testing for disability-related diseases if it were available in a pharmacy?	Highly likely	182	38.3
	Maybe	233	49.1
	No at all	60	12.6
Do you support pharmacies conducting genetic testing for disability-causing diseases?	Yes	302	63.6
	No	72	15.2
	Not sure	101	21.3

* Multiple-answer questions result in percentages exceeding 100%.

**Table 7:** Comparison of domain scores across demographics.

Demographics	Total	Knowledge score	Perception score	Acceptance score	Demographics
Age	18–24	160	4.74 ± 1.3^A^	7.58 ± 2.2^A^	12.12 ± 2.0^AB^
	25–31	27	4.48 ± 1.2^AB^	7.04 ± 2.9^AB^	11.15 ± 2.9^A^
	32–38	37	5.11 ± 1.2^A^	6.54 ± 2.4^AB^	12.92 ± 1.7^B^
	>38	251	4.46 ± 1.3^B^	5.98 ± 2.6^B^	11.73 ± 2.4^A^
*P*-value			.011*,†	<.001*,‡	.004*‡
Gender	Male	276	4.47 ± 1.3	6.29 ± 2.6	11.79 ± 2.3
	Female	199	4.79 ± 1.2	7.08 ± 2.5	12.10 ± 2.2
*P*-value			.008§	<.001§	.150
Highest educational level	Less than secondary school	17	3.94 ± 1.2^A^	5.00 ± 2.6^A^	11.94 ± 2.7
	Secondary school	140	4.69 ± 1.3^B^	6.57 ± 2.4^B^	11.75 ± 2.1
	University degree	268	4.48 ± 1.2^AB^	6.65 ± 2.6^B^	11.99 ± 2.2
	Postgraduate studies	50	5.28 ± 1.4^C^	7.16 ± 2.3^B^	12.00 ± 2.5
*P*-value			<.001*,†	.027*,†	.769
Do you have a background in healthcare or related fields?	Yes	315	4.99 ± 1.2	6.95 ± 2.6	12.08 ± 2.2
	No	160	3.84 ± 1.0	5.98 ± 2.4	11.61 ± 2.4
*P*-value			<.001§	<.001§	.031§
Have you or any of your family members been diagnosed with a disease that may lead to disability?	Yes, I have been diagnosed	6	5.50 ± 1.4^AB^	5.00 ± 2.3	12.00 ± 2.8
	Yes, a family member has been diagnosed	29	5.14 ± 1.3^B^	7.03 ± 2.5	11.86 ± 2.7
	No	426	4.56 ± 1.3^A^	6.61 ± 2.6	11.92 ± 2.2
	Not sure	14	4.43 ± 1.1^AB^	6.93 ± 2.4	12.07 ± 2.1
*P*-value			.035*,†	.336	.993

Capital letters represent the post hoc multiple pairing summary indicator. When 2 measures share the same letter, they are statistically similar.

* Significant using 1-way ANOVA test at < 0.05 level.

† Post hoc test = LSD.

‡ Post hoc test = Games-Howell.

§ Significant using independent *t*-test at < 0.05 level.

**Figure 3.**
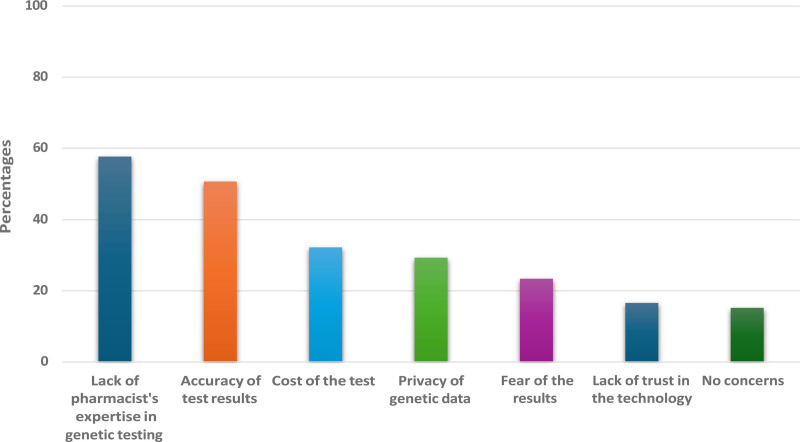
Concerns about pharmacy-based genetic testing.

The assessment of reliability is presented in Table 4, with moderate reliability observed in the Knowledge (α = 0.581) and Acceptance (α = 0.636) domains. The Perception domain, however, showed lower internal consistency (α = 0.467). Construct validity was supported by a moderate positive correlation between Perception and Acceptance scores (*R* = 0.214, *P *< .001), suggesting a link between favorable perceptions and higher acceptance of genetic testing in pharmacies as illustrated in Table 6. The average scores for the Knowledge, Perception, and Acceptance domains were 4.61, 6.62, and 11.92, respectively, as shown Table 5, These values reflect moderate levels of awareness, a cautious but positive perception, and an overall openness toward acceptance of genetic screening in pharmacy settings. Further construct validity is supported by Table 6, where correlations reveal that higher Perception scores correlated with increased Acceptance (*R* = 0.214, *P *< .001), suggesting that positive perceptions align with greater acceptance. A weaker correlation between Knowledge and Perception (*R* = 0.114, *P *= .013) indicates that while knowledge plays a role, it may have less direct influence on comfort and acceptance. Table 7 explores differences in Knowledge, Perception, and Acceptance scores across demographic groups. Younger participants (18–24 years) reported significantly higher Perception scores than older age groups (*P *< .001). Females scored higher in both Perception and Acceptance domains than males, and those with healthcare backgrounds scored higher across all domains, underscoring the impact of age, gender, and healthcare experience on attitudes toward genetic testing. Concerns about pharmacy-based genetic testing are shown in Figure 3. Regression analyses, summarized in Table 8, examined how demographic factors predict Knowledge, Perception, and Acceptance scores. Age, educational level, and healthcare background were significant predictors. Participants aged 32 to 38 exhibited higher Acceptance scores (B = 1.072, *P *= .008), while higher educational attainment and healthcare experience were strongly associated with increased Knowledge and favorable Perception scores.

**Table 4:** Reliability analysis (Cronbach alpha).

Reliability statistics	Cronbach alpha	No. of items
Knowledge score	0.581	3
Perception score	0.467	3
Acceptance score	0.636	5

**Table 5:** Domain scores for knowledge, perception, and acceptance.

Domains	N	Min	Max	Mean	SD
Knowledge score	475	3.0	8.0	4.61	1.3
Familiarity with genetic screening	475	1.0	3.0	1.67	0.6
Previous experience with genetic testing	475	1.0	2.0	1.27	0.4
Self-rated knowledge of genetic testing	475	1.0	3.0	1.67	0.6
Perception score	475	1.0	13.0	6.62	2.6
Perceived benefits of genetic screening	475	0.0	4.0	2.37	1.3
Comfort with genetic screening in a pharmacy	475	1.0	3.0	2.25	0.7
Concerns about genetic screening in pharmacies	475	0.0	6.0	2.00	1.5
Acceptance score	475	5.0	15.0	11.92	2.3
Willingness to undergo genetic testing if available in a pharmacy	475	1.0	3.0	2.26	0.7
Preference for counseling (pharmacist vs specialist)	475	1.0	3.0	1.81	0.9
Likelihood of taking preventive measures if at-risk	475	1.0	3.0	2.68	0.6
Support for pharmacies conducting genetic testing for disability-causing diseases	475	1.0	3.0	2.48	0.7
Preference for pharmacy and specialist collaboration	475	1.0	3.0	2.69	0.6

**Table 6:** Construct validity through domain correlations.

Correlations		Perception score	Acceptance score
Knowledge score	*r*	0.114*	0.063
	*P*-value	.013	.168
	N	475	475
Perception score	*r*		0.214**
	*P*-value		<.001
	N		475

* Correlation is significant at the 0.05 level (2-tailed).

** Correlation is significant at the 0.01 level (2-tailed).

**Table 8:** Parameter estimates for predictive modeling of domain scores.

Dependent variable		B	SE	95% confidence interval		*P*-value
				Lower bound	Upper bound	
Knowledge score	Intercept	4.384	0.366	3.664	5.103	<.001*
	Age					
	18–24	0.044	0.135	−0.221	0.309	.744
	25–31	−0.149	0.234	−0.609	0.312	.527
	32–38	0.371	0.206	−0.033	0.776	.072
	Gender					
	Male	−0.212	0.113	−0.433	0.010	.061
	Highest educational level					
	Less than secondary school	−0.669	0.334	−1.325	−0.012	.046*
	Secondary school	−0.385	0.207	−0.792	0.022	.064
	University degree	−0.525	0.183	−0.885	−0.165	.004*
	Do you have a background in healthcare or related fields?					
	Yes	1.014	0.118	0.782	1.246	<.001*
	Have you or any of your family members been diagnosed with a disease that may lead to disability?					
	Yes, I have been diagnosed	0.805	0.568	−0.311	1.920	.157
	Yes, a family member has been diagnosed	0.601	0.375	−0.136	1.339	.110
	No	0.027	0.315	−0.591	0.646	.931
Perception score	Intercept	7.070	0.767	5.563	8.576	<.001*
	Age					
	18–24	1.680	0.282	1.125	2.234	<.001*
	25–31	0.873	0.491	−0.092	1.837	.076
	32–38	0.198	0.431	−0.649	1.044	.646
	Gender					
	Male	−0.267	0.236	−0.731	0.196	.258
	Highest educational level					
	Less than secondary school	−2.147	0.699	−3.520	−0.773	.002*
	Secondary school	−1.428	0.433	−2.279	−0.576	.001*
	University degree	−0.781	0.384	−1.536	−0.027	.042*
	Do you have a background in healthcare or related fields?					
	Yes	0.502	0.247	0.016	0.988	.043*
	Have you or any of your family members been diagnosed with a disease that may lead to disability?					
	Yes, I have been diagnosed	−1.481	1.188	−3.816	0.855	.213
	Yes, a family member has been diagnosed	0.152	0.786	−1.392	1.696	.847
	No	−0.344	0.659	−1.640	0.951	.602
Acceptance score	Intercept	11.846	0.714	10.444	13.248	<.001*
	Age					
	18–24	0.380	0.263	−0.136	0.897	.148
	25–31	−0.667	0.457	−1.565	0.231	.145
	32–38	1.072	0.401	0.284	1.860	.008*
	Gender					
	Male	−0.192	0.220	−0.623	0.240	.384
	Highest educational level					
	Less than secondary school	0.232	0.651	−1.046	1.511	.721
	Secondary school	−0.291	0.403	−1.083	0.502	.471
	University degree	0.069	0.357	−0.633	0.771	.847
	Do you have a background in healthcare or related fields?					
	Yes	0.358	0.230	−0.094	0.810	.121
	Have you or any of your family members been diagnosed with a disease that may lead to disability?					
	Yes, I have been diagnosed	0.217	1.106	−1.956	2.390	.845
	Yes, a family member has been diagnosed	−0.186	0.731	−1.623	1.251	.800
	No	−0.199	0.614	−1.405	1.007	.746

* Significant using general linear model at <0.05 level.

## 4. Discussion

Pharmacies are widely considered as easily accessible, and they frequently serve as the primary point of contact for patients seeking medical counsel and services. However, there is a global lack of implementation of genetic testing in community pharmacies, especially for disability-related diseases.^[12–15]^ Therefore, this study aimed to assess public perceptions of genetic testing for disability-related diseases, particularly in Saudi Arabian pharmacies.

### 4.1. Clients

The findings indicated that the majority (around 87%) of the participants were over 18% and 58% of them were male, which might affect the generalizability of the results. While the data indicated that the participants were well educated, with 56% holding a university degree and 66% having a healthcare-related background, the sample was not representative of the broader population’s educational distribution, which is likely to have affected their understanding of genetic testing.

### 4.2. Perceptions of genetic testing

Overall, the respondents considered their knowledge and awareness of genetic testing as limited, with only 9% identifying themselves as very knowledgeable. Notably, 84.4% of participants recognized that early detection and diagnosis were important. This indicates that genetic testing is crucial as a potential preventive measure for disability-related diseases.

Around 42% of the participants expressed comfort with undergoing genetic testing in pharmacies, indicating a general acceptance of genetic testing in public pharmacies. A significant gender difference was observed, with female participants expressing higher levels of comfort and acceptance compared to males. However, there was considerable uncertainty among the participants, with about 40% being unsure about the accuracy of the test, highlighting potential concerns or lack of information about genetic testing. Around 18% of the participants were clearly not convinced about the use of genetic tests, suggesting some resistance, possibly due to fears of the results, stigma, or misconceptions about genetic testing in relation to diseases that are associated with disabilities.

Acceptance levels were generally positive, with most participants supporting the introduction of genetic testing in pharmacies. However, their willingness to undergo testing was significantly influenced by demographic factors such as age, gender, education level, and healthcare background. Younger participants and those with healthcare-related education displayed higher acceptance levels, underscoring the importance of targeted awareness campaigns for older and less-educated demographic groups. Additionally, individuals with a healthcare background scored higher in the knowledge, perception, and acceptance domains, emphasizing the positive impact of professional exposure on shaping attitudes toward genetic testing.

### 4.3. Perceptions of pharmacists’ competency

The study also revealed mixed public perceptions regarding pharmacy-based genetic testing. While many participants recognized convenience (76%) and cost-effectiveness (45%) as motivating factors, a significant concern raised about the lack of pharmacist’ expertise in genetic testing (58%) and test accuracy (51%). Moreover, 51% of participants prefer to receive consultation from a specialist physician or collaborate with them (76%) following the screening, indicating a desire for expert guidance in interpreting results. These insights emphasize a trust gap in pharmacist professional capabilities to deliver genetic results to patient. Similar outcomes were reported in another study conducted in England and Beirut which showed that the community pharmacy is not prepared to deliver a genetic rest.^[12,16]^

Additionally, around 51% are concerned about the accuracy of test results, highlighting the need for reliable testing methods. Other noted concerns include the cost of testing (32%), privacy of data (29%), and fear of the test results (23%). Despite these concerns, 15% of participants have no concerns, indicating a small percentage of the population trusts the healthcare process. Previous research suggests that the outcomes of genetic tests may vary based on their source, therapy raise the concern of accuracy and whether the is provide comprehensive knowledge.^[17,18]^

## 5. Conclusion

This study provides valuable insights into public knowledge, perceptions, and acceptance of genetic testing for disability-related diseases in pharmacies across Saudi Arabia. While public acceptance of pharmacy-based genetic testing was generally positive, significant knowledge gaps and trust issues remain, particularly concerning pharmacist expertise and the accuracy of test results. Addressing these gaps through targeted public education campaigns, pharmacist training programs, and structured collaborative care models with healthcare specialists is essential. These measures will not only enhance public trust but also ensure the successful integration of genetic testing services into pharmacy practice.

## 6. Limitations

The main limitation of this study is the use of convenient sampling which may limit the generalizability of the findings to the wider population in Saudi Arabia. While the study valuable, there is a concern of self-reported data as may introduce biases such as social desirability and recall errors, potentially affecting the accuracy of the results. Additionally, the study focused exclusively on pharmacies, which may not fully capture public perceptions of genetic testing in other healthcare settings. Furthermore, variations in participants’ familiarity with digital health tools and technologies might have influenced their responses. Future research should address these limitations by employing larger, randomized samples and longitudinal study designs to observe changes in public perceptions over time. Additionally, qualitative approaches could offer deeper insights into the underlying reasons for public acceptance or hesitation toward genetic testing in pharmacy settings.

## Acknowledgments

The authors extend their appreciation to the King Salman Center For Disability Research for funding this work through Research Group no. KSRG-2024-294.

## Author contributions

**Conceptualization:** Saad M. Wali.

**Formal analysis:** Mohammed M. Aldurdunji.

**Resources:** Reem Hasaballah Alhasani, Shaker T. Alsharif.

**Software:** Mustfa Faisal Alkhanani, Mohammed S. Alharthi, Nasser M. Aldekhail.

**Supervision:** Ahmed M. Ashour.

**Validation:** Nasser M. Alorfi, Ahmed M. Ashour, Ohood K. Almuzaini.

**Visualization:** Nasser M. Alorfi, Fahad S. Alshehri, Ohood K. Almuzaini.

**Writing – original draft:** Nasser M. Alorfi, Ahmed M. Ashour, Fahad S. Alshehri, Maan H. Harbi.

**Writing – review & editing:** Nasser M. Alorfi, Ahmed M. Ashour, Fahad S. Alshehri, Maan H. Harbi.
